# A Case of Hydrophobia

**Published:** 1794

**Authors:** Richard Simmons

**Affiliations:** Surgeon to the British Lying-in Hospital.


IX. *A Cafe of Hydrophobia. By Mr.* Richard Simmons, *Surgeon to the Britifh Lying-in Hofpital.*

ON Friday, Auguft the 23d, 1793, about one o'clock in the afternoon, I was requefted to fee Mary Strong, a poor woman, at No. 11, Lewkner's Lane, aged forty-three years. The account fhe gave me was, that fhe had been unable to fwallow any liquids during the laft two days, as fhe no fooner put fome to her mouth, than fhe was feized with a kind of fuffocation. She obferved, however, that fhe could fwallow folids. She had, I was told, eaten an apple very well juft before I faw her, and in my prefence fhe ate fome bread.

She was coſtive; her pulſe was natural; but her thirſt was extremely diſtreſſing. I deſired ſhe would try to drink ſome water, and ſhe accordingly made the attempt; but no ſooner had ſhe brought the pot containing ſome near her lips, than ſhe threw her head back with great agitation, and could not get any into her mouth. I then aſked for a pan of water, which I put up to her face; this produced in her very great emotion. A woman, who was preſent, thought this agitation might ariſe from an idea that the water was going to be thrown at her; but the patient's manner being ſufficient to convince me there was no ground for ſuch a ſuppoſition, I aſked her to approach her face to it gently, when placed near her. This ſhe very readily tried to do, but the event was the ſame as when I held the pan.

From theſe circumſtances I had no doubt that ſhe laboured under hydrophobia; and upon farther inquiry, I learnt that ſhe had been bitten about two months before, by a dog, on the middle finger of her left hand. The wound being trifling, ſhe had thought no more of it; the dog, I was informed, had been killed by ſome people in the neighbourhood, on a ſuppoſition of its being mad.

I directed for her opium, in fubftance, to be occafionally repeated; but the next day (the 24th) when Dr. Hemming accompanied me to her, we found that fhe had refufed to take any thing in the way of medicine, but that fhe had taken often of fome bread foaked in wine and water. She had paffed but an indifferent night, and her pulfe was much quickened. Upon our requefting her to endeavour to drink fomething, fhe told us fhe would, if poffible, do any thing we defired of her; but that although her thirft was ftill exceffive, it was not in her power to drink. We advifed her to try to drink fomething out of a tea pot, but the attempt threw her into great diftrefs. After we had been with her a little while, however, fhe fwallowed a tea fpoonful of wine and water in a hafty confufed manner. This induced us to perfuade her to try to take a larger draught; fhe took fome in a tea cup in her hand, and threw it into her mouth haftily, and with great difficulty fwallowed a very little of it; but immediately after threw herfelf back in the bed in a convulfive kind of manner.

We now left her, and advifed that fhe fhould have a clyfter injected immediately; and that an ounce of ung. hydrarg. fort. fhould be rubbed bed in on her thighs during the day; ſhe was likewiſe adviſed to make trial of the opiate.

I ſaw her again about half paſt twelve o'clock on the 25th. She had had no ſleep, talked in an incoherent manner, hiccoughed, and ſpate frequently a viſcid ſaliva, and foamed at the mouth; her pulſe was now not perceptible; the clyſter had produced one copious ſtool; ſhe had been unable to take any of the opiate, and had refuſed to have more than two drachms of the ointment rubbed in. Her countenance was wild; her ſpeech altered; and every appearance indicated a ſpeedy diſſolution, which accordingly took place that afternoon, about three o'clock, two hours after I left her. From the people who were about her, I afterwards learnt that ſhe died very quietly, and that juſt before her death ſhe vomited about a pint of ſomething, as they deſcribed it, like coagulated blood.

Leave could not be obtained to open the body.

There is ſo little to be deduced from this caſe, that ſome apology might be neceſſary for offering it to the public, if the diſeaſe of which it it is an inftance, were lefs rare or better underftood; but in the prefent imperfect ftate of our knowledge with refpect to the pathology and treatment of hydrophobia, every well-authenticated inftance of it feems to deferve to be recorded. Some valuable obfervations refpecting this difeafe, by Dr. John Hunter, drawn up from cafes and materials collected by a Society for the improvement of medical and chirurgical knowledge, have been lately publifhed in their Tranfactions; and a fimilar arrangement of facts, made from time to time, may perhaps, by degrees, lead us to a more fuccefsful mode of treating a difeafe which has hitherto baffled every attempt to relieve it.

The little deviation from the natural ftate of the pulfe, which occurred during the firft two days in my patient, has been obferved in other inftances at the beginning of the difeafe.

In this cafe, notwithftanding her inability to fwallow any thing liquid, the patient, as we have feen, complained of very diftreffing thirft; a fymptom which I do not find mentioned in the defcriptions I have met with of other inftances of the difeafe.

